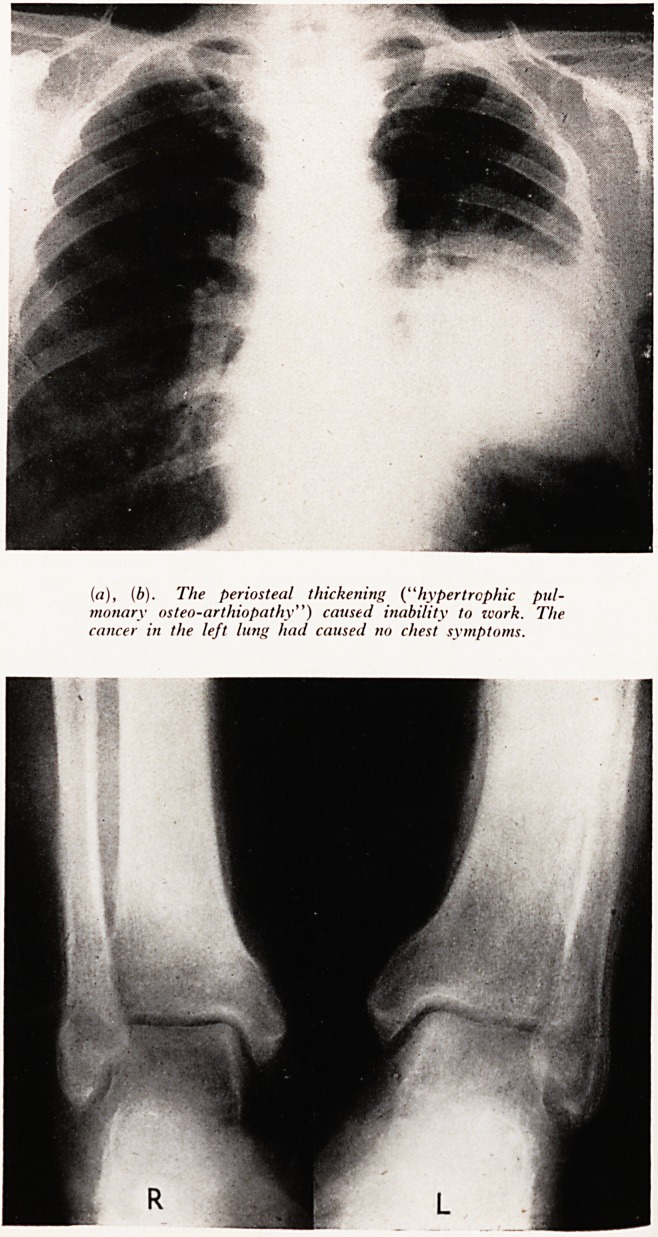# Lung Cancer—A Symposium

**Published:** 1959-07

**Authors:** J. E. G. Pearson

**Affiliations:** Consultant Physician, United Bristol Hospitals and Frenchay Hospital


### LUNG CANCER—A SYMPOSIUM

### PART I—A General Review

BY

J. E. G. PEARSON, M.D., F.R.C.P.

*Consultant Physician, United Bristol Hospitals and Frenchay Hospital*

In opening this evening's symposium, I trust we can all agree that without doubt lung cancer has shown a true rise in incidence in the past quarter of a century. Improved diagnosis has certainly played its part but, in my opinion, cannot account for anything like the great increase which is apparent during this time. In 1930 bronchial carcinoma accounted for about 2 per cent. of all cancer deaths; by 1950 this figure had risen to 20 per cent. The Regional Cancer Bureau now tells us that bronchial neoplasms are commoner than any other, forming about 10 per cent. of all cancers.* Herein lies the challenge to us all.

The few figures which I shall produce to illustrate the subject are the average of a number of publications including the largest series of over a thousand patients analysed by the British Empire Cancer Campaign and some 280 patients under my own care.

The peak of age incidence occurs around 54 years but no age group is exempt, the youngest reported being 4 years and the oldest 100. Males suffer more than females in the proportion of at least 6 to 1 though possibly this difference is slowly lessening.

Concerning aetiology, I do not propose to open up the subject of the possible relationship of cigarette smoking to cancer. This has received quite enough fiery attention in the lay and medical press. Whatever the truth, we must be prepared to diagnose the condition in non-smokers, as were in fact the first three cases I saw after the real publicity drive started. Nor must we forget the importance of finding out what the relationship of atmosphere pollution may be to this condition.

One small point I would like to make about cigarette smoking is that it renders cough virtually valueless as a symptom by which we are to spot the early carcinoma. The smoker's cough merges so easily into the carcinoma cough—too gradually for the patient to appreciate it until too late.

### SYMPTOMS

Our main concern is to arrive at an early diagnosis of a condition which may be so insidious in onset.

Cough and sputum themselves are unlikely to be very helpful symptoms for the reason I have just mentioned. Brassy cough previously emphasised is not perhaps such an early symptom as was thought because it may imply there is already mediastinal involvement. Shortness of breath for no other demonstrable reason must arouse suspicion; it can occur because a bronchus is fixed by growth before any lung collapse takes place. Pain in the chest, usually a late symptom due either to pleural or chest wall involvement, can also be an early one on occasion, localised over a small area of pulmonary collapse. Haemoptysis will always bring the patient to the doctor and lucky may be the man whose bronchial carcinoma bleeds early. Loss of weight is a late symptom and usually occurs only after matastasis has taken place.

I would say that most of the *early* cases are picked up in one of three ways: First, haemoptysis; second, a pneumonic type of attack where the affected part of the lung does not fully clear either clinically or radiologically or both—the "unresolved pneumonia" of former days. Thirdly, routine X-ray. In the first three-quarters of a million mass X-rays based on Bristol only one case of lung cancer was picked up in every 4,500. The incidence of cases sent by general practitioners to the static X-ray unit is around one in 300, showing how well aware these doctors are of this problem, and I can only exhort them to have even more chest X-rays performed on even slighter suspicion in future.

* Carcinoma of the breast in females alone is slightly commoner than carcinoma of the bronchus in males alone.

Apart from the evidence of bronchopulmonary infection which I have already mentioned, signs may be remarkable by their absence in the early stages before any bronchial obstruction has taken place. Sometimes evidence of bronchial narrowing may be obtained by the presence of weaker air entry to a lobe or segment, or of a "fixed" rhonchus over one area—one which cannot be coughed away—but it is only occasionally the stethoscope will be really more revealing than the X-ray as in the following case (Plate XXVI).

This man, aged 69, was complaining of severe shortness of breath and slight haemoptysis. His X-ray showed rather a heavy left hilar shadow and suggested collapse of the left lower lobe. Auscultation revealed almost complete absence of breath sounds over the whole of the left lung and urgent bronchoscopy showed, as I suspected, his left main bronchus was almost completely occluded by growth and the whole lung about to collapse.

### METASTASES

In trying to assess whether a patient has a growth of early type, we should of course know where we may particularly expect metastases. Table I gives an idea of this and the figures are, of course, post mortem ones. I would point out one notable omission—

TABLE I

Showing the site of metastases from bronchial carcinoma.
(727 cases : 636 autopsies)

| | *Per cent* |
|---|---|
| Intrathoracic glands | 90 |
| Adrenals | 41 |
| Other lung | 40 |
| Abdominal glands | 40 |
| Liver | 35 |
| Kidneys | 24 |
| Bones | 21 |
| Brain | 18 |
| Extension to heart muscle | 9 |

that of glands around the thorax, that is superclavicular or axillary, which are fairly commonly involved. Although 10 per cent. are said not to have had any metastasis by lymphatic spread to the mediastinum, we must remember that this apparently favourable type of growth, probably mostly peripheral in site, may nonetheless metastasise early through the blood stream.

I would also draw your attention to the high incidence of involvement of the abdominal glands usually around the coeliac axis and often involving the pancreas. These may cause symptoms such as vomiting, or haematemesis, and be mistaken for gastric carcinoma especially if a mass is felt.

One other site in which I have been especially interested is the spread to the heart muscle and of course pericardium. An auricle is usually involved, in 9 per cent. of cases as you see, and results often in an arrhythmia such as auricular fibrillation; this may indeed by the presenting sign.

There are other unusual methods of presentation: bleeding from the rectum I have seen from a secondary deposit in the sacrum, thrombocytopenic purpura in a man of 80 due to bone marrow invasion, "arthritis" so-called, really pulmonary hypertrophic osteoarthropathy which is fairly common even without much associated sepsis in the chest; and perhaps strangest of all, a girl of 21 with erythema nodosum who at autopsy had a large anaplastic bronchial carcinoma with no evidence of tuberculosis anywhere.

### DIAGNOSIS

Normally we hope to arrive at an early diagnosis firstly by being suspicious of symptoms however vague and whether supported by physical signs or not; secondly by X-ray findings, including tomograms: there again, suspicion of any odd shadow in the lung plays its part. In fact, the absence of any radiological abnormality does not exclude a growth; I have seen a normal X-ray in a man who had intense dyspnoea and bronchoscopy revealed growth invading both main bronchi. Thirdly, bronchoscopy should give direct or indirect evidence of growth in the majority of cases, but by no means all of these give a positive biopsy. In the remainder and where findings are negative, we do not here recommend routine search of sputum for malignant cells; for the results achieved, the procedure is extremely time-absorbing for the pathologist and obviously many false negatives will be found. We consider it much safer in the doubtful case and, mark you, just the sort of case that may be curable, to proceed direct to exploratory thoracotomy.

### DIFFERENTIAL DIAGNOSIS

This can raise difficult problems and I cannot now enlarge much upon them. I would say that the main differentiation is to be made from tuberculosis, bronchiectasis and lung abscess; not infrequently however, a carcinoma may co-exist with any one of these. A less well appreciated differential diagnosis is heart failure—shortness of breath, pleural effusion, perhaps haemoptysis and cervical vein engorgement which might be thought due to mediastinal obstruction. Very occasionally an unexpected foreign body will account for unexplained collapse of a lobe. Otherwise differential diagnosis is mainly a radiological affair.

### PROGNOSIS

The average survival of untreated cases is only 10 months from the time of the first symptom, though some patients with squamous growths may live upwards of two years. Histologically, squamous cell growths form about a third of the total, some of course being more differentiated than others. They offer the best chance of a cure and the great majority of surgical successes lie in this group.

Surgery is the treatment of choice. It is the overall results which are so disappointing because until recently less than 40 per cent. of all cases were found worth exploring by thoracotomy and under 25 per cent. proved resectable. Many of these will already have metastasised beyond surgical bounds to recur later in one of the sites I have mentioned. The 5-year cure rate, which very closely resembles the 3-year cure rate, is only about 6 per cent. of *all* cases. No wonder we are depressed at this figure and no wonder we continue to encourage every effort towards early diagnosis of this very insidious disease.

About deep X-ray therapy I would like to make one point. Remembering that the usual type of case referred to the radiotherapist has much the worst prognosis and life can seldom be prolonged for more than a few weeks by that means, I think it is the duty of us all to resist pressure from anxious relatives for radiotherapy in order to feel that "everything is being done". This attitude is not fair to the patient nor to the radiotherapist and we should be allowed to adhere to our genuine indications.

I find that most of the patients whom I refer with a view to radiotherapy fall into the following groups, all of them being clearly unsuitable for operation by reason either of their local or general condition:

(1) Those with mediastinal obstruction.

(2) Those with severe pain from involvement of the thoracic cage by the primary growth (e.g. "Pancoast tumour") or by metastasis elsewhere (e.g. spine); even so, the palliative results may be disappointing in this group.

(3) When there is imminent threat of complete obstruction of a main bronchus.

(4) A variety of cases in which, after consultation with the radiotherapist, it is decided that the growth seems to be sufficiently well localized to recommend a trial of radical radiotherapy; implicit in this decision is the failure to demonstrate any suggestion of extrathoracic metastasis.

### PART II

### Radiology

BY

K. H. GASKELL, M.A., B.CHIR., D.M.R.D.
*Consultant Radiologist, Frenchay Hospital*

X-ray of the chest is usually the first investigation needed in patients with chest symptoms. But the evidence gathered from these X-rays should be regarded as a supplement to the evidence already collected by the clinical examination—by inspection, palpation, percussion and auscultation—and a film may or may not show an abnormal shadow even when a growth is present.

It is true that serial X-rays may reveal the development and spread of a growth but further investigation such as bronchoscopy should not be delayed while diagnostic X-ray shadows are awaited, for by so doing we are conducting a radiological requiem in a previously treatable and perhaps curable case.

The following X-ray films are of cases which are grouped together according to the similarity of their shadows and their mode of presentation.

### GROUP I

The first two cases are of proved bronchial carcinoma with very little radiological evidence of the disease.

Plate XXVII (*a*). (Male aged 62.) There is no abnormal shadow. At thoracotomy and left pneumonectomy the subaortic and hilar lymph nodes were enlarged, and on section the hilar nodes were invaded by metastases of squamous carcinoma.

Plate XXVII (*b*). (Male aged 59.) The X-ray is suspicious but not conclusive of abnormality above the left lung root. At operation a squamous growth was removed and this had involved the hilar glands. The patient three months before had a pneumonia-like illness with blood streaking of sputum and chest pain which continued until operation.

These two cases fortunately received effective surgical treatment without waiting for positive X-ray signs of growth to develop.

### GROUP II

We will now consider the outcome of cases having more pronounced shadows. These two cases also show the contrast between X-ray findings and clinical state.

Plate XXVIII (*a*). (Male aged 42.) Noticed a wheeze in December 1955, which gradually lessened but was replaced by shortness of breath. A sudden left chest pain followed in February 1956, which precipitated investigations. X-ray shows occlusion of the left main bronchus. Through the examining bronchoscope pieces of a soft fungating growth were removed with forceps, and the following day (Plate XXVIII (*b*)) the chest film shows complete re-expansion and no shadows of growth. Following pneumonectomy and clearance of interbronchial glands (oat-cell carcinoma) the man has enjoyed a useful life and a year later moved to Manchester.

Plate XXIX (*a*) (Male aged 49). The X-ray shows a small shadow in the right upper hilar region which at thoracotomy proved to be a completely inoperable growth extending backwards into the mediastinum.

Decision as to operability can seldom if ever be made from the X-ray.

### GROUP III

These next two cases illustrate unusual forms of presentation. Plate XXIX (*b*) (Female aged 19.) She had chest pain dyspnoea and cough increasing for 6 months before thoracotomy. Tuberculosis, and later sarcoidosis, were at first suspected to be

*Chest X-ray of a man aged 69. The left hilar shadow is heavy. On physical examination, breath sounds were absent over the left lung. On bronchoscopy the left main bronchus was almost occluded by growth.*

(*a*) *At operation the left lung was removed and on section metastases were found in the left hilar lymph nodes.*

(*b*) *There is a suspicious shadow at the left lung root. At operation hilar lymph nodes were involved by growth.*

PLATE XXVIII

(*a*) *Collapse of the left lung.*

(*b*) *Re-expansion following removal of pieces of growth obstructing the left main bronchus. This patient is still alive and active after pneumonectomy.*

PLATE XXIX

(*a*) *There is only a small shadow in the right hilum but at operation the growth was quite inoperable.*

(*b*) *Tomography in a girl of* 19, *showing a growth in the right stem bronchus. Clinically tuberculosis and sarcoid were suspected.*

(*a*) *This shows old quiescent tuberculosis and also an area of collapse due to a squamous-cell carcinoma in the right upper lobe.*

(*b*) *The peripheral carcinoma in the left lung was symptomless; the patient presented with symptoms from cerebral metastases.*

(*a*), (*b*). *The periosteal thickening ("hypertrophic pulmonary osteo-arthiopathy") caused inability to work. The cancer in the left lung had caused no chest symptoms.*

the cause but bronchoscopy revealed growth in the stem bronchus. The tomogram film reproduced here shows these features well. The case was inoperable, growth having invaded the pericardium.

Plate XXX (*a*). (Male aged 56.) This man was under observation for quiescent tuberculosis when a second lung shadow of segmental right upper lobe collapse developed during an influenza-like illness. Thoracotomy confirmed the dual pathology and resection of a squamous growth without glandular involvement was carried out. Brain metastases however, developed 6 months later.

### GROUP IV

This group are examples of bronchial carcinomata that were silent. Patients frequently present first with metastases in brain or skeleton in whom a primary asymptomatic growth is present in the lung.

Plate XXX (*b*). (Male aged 49.) Following an attack of paraesthesia in the right hand, this man had a fit and further cerebral localizing signs. Investigations revealed a small left lung peripheal shadow, and glands in the interbronchial and paratracheal positions. Cerebral biopsy confirmed an adenocarcinomatous deposit in the left hemisphere.

Plate XXXI (*a* and *b*). (Male aged 52.) A coalminer fractured his left tibia at work 8 months before an insurance assessment. He rightly stated his inability to work, but complained of no chest symptoms. Both leg films show diffuse periosteal proliferation and, in the chest film, there is a massive peripheral shadow. The periosteal change is that of a well-marked hyertrophic pulmonary osteopathy associated with lung disease.

### SUMMARY

These X-rays serve to show how radiology of the chest in cancer patients must be integrated with the patient's history, physical signs, bronchoscopy and exploration of the chest.

### LUNG CANCER—A SYMPOSIUM

### Part III—Surgical Treatment

BY

R. H. R. BELSEY, M.S., F.R.C.S.

*Consultant Thoracic Surgeon, United Bristol Hospitals and Frenchay Hospital*

The results of treatment for cancer of the lung can cause nothing but gloom. The results of a personal series of cases treated in the South-West Regional Thoracic Unit are summarised briefly in Table II. The overall five-year survival rate for the whole

TABLE II

*Bronchial carcinoma*

1942–1957 = South-West Regional Thoracic Unit at Frenchay Hospital.
1,239 cases admitted to Surgical Unit
625 inoperable on bronchoscopy
182 inoperable at thoracotomy
432 resected
Overall 5 year survival rate (1945–51) = 5 per cent.
(Whole South-West Region)

South-West Region from 1945-1951 is only 5 per cent. The reason is delay in diagnosis and in treatment, rather than any unusual or excessively macabre feature of this disease. The only method of cure known at present is surgical resection. When this operation can be performed at all, (for the most part for advanced disease with extensive involvement of hilar and mediastinal lymph nodes), the five year survival rate is 31.8 per cent. which is better than the corresponding figures for cancer of the bladder, skeleton, larynx, stomach, kidney or prostate. (See Table III).

TABLE III

*Survival rate following resection*

Of the 432 lung resections for carcinoma the 5 year survival rate for resected cases has been 31·8 per cent., compared with the following results in some other sites for carcinoma:

| | |
|---|---|
| Bladder .. .. .. .. | 31 per cent |
| Bones .. .. .. .. | 28·5 per cent |
| Larynx .. .. .. .. | 23 per cent. |
| Stomach .. .. .. .. | 21 per cent. |
| Kidney .. .. .. .. | 20 per cent. |
| Prostate .. .. .. .. | 15 per cent. |

Despite the fact that bronchial carcinoma is still a "surgical disease" probably not more than 50 per cent. of cases are ever seen by a surgeon. This implies that decisions on operability are being made elsewhere. Obvious metastases in the C.N.S., skeleton, or liver, exclude all consideration of surgical treatment. Enlarged lymph glands, possibly metastatic, merit a surgical opinion. But any decision on operability based upon radiological changes, bronchoscopic appearances, and the general condition of the patient, must be the responsibility of the surgeon who is going to perform the operation.

### SURGICAL PROGRESS

Surgical technique advances and with this progress there is an increase in the operability rate in every form of malignant disease. The resection rate has risen from 10·4 per cent. in 1946 to 52·2 per cent. in 1956, and these figures afford a little encouragement in an otherwise gloomy situation. Both surgeons and anaesthetists have abandoned the traditional criteria of a patient's ability to withstand major surgery. Very few patients have been denied surgery on account of their poor general condition, which is so frequently due to the septic complications of bronchial obstruction. Previous cardiovascular catastrophies and lurid E.C.G. pictures do not preclude successful pulmonary surgery. In a personal series of 432 resections, many carried out by a registrar or house surgeon, the mortality rate has been 7·7 per cent. which compares favourably with published rates ranging from 11 per cent to 25 per cent. This figure can be attributed largely to good anaesthesia of the simple old-fashioned type, good physiotherapy and nursing, and the vigilance of the resident staff.

The surgical technique of pulmonary resection is now fairly well standardised. The intra-pericardial dissection of the lung root permits a more radical resection, including involved portions of the left atrium. Partial resection and reconstruction of the trachea is possible. A block dissection of enlarged mediastinal lymph glands is carried out routinely but often the enlargement is found to be inflammatory rather than neoplastic. Large sections of the chest wall involved by a peripheral carcinoma can be resected along with the lung, and reconstruction effected by stainless steel wire mesh. The post-operative morbidity rate is low and the more serious complications such as bronchial fistulae, pleural infection and contra-lateral lung infection have now been largely controlled.

Lung resection has become both more radical and more conservative. For hilar growths the radical pneumonectomy already described is performed. For peripheral growths a simple lobectomy is adequate. The long term results are as good and the functional results better than for total lung resections, owing to the lesser degree of compensatory emphysema that develops in the remaining lung tissue. About a third of all lung resections for cancer are now limited to one lobe, but earlier diagnosis may justify an even higher percentage of partial lung resections.

### CONTRA-INDICATIONS TO OPERATIONS

There are a few indefinite contra-indications to lung resection. Patients over 70 do not tolerate pneumonectomy well; on the other hand, patients of 75 and over may stand a lobectomy without difficulty. Gross contra-lateral emphysema may be a contra-indication but it is difficult or impossible to assess residual lung function from X-ray appearances. Gross chronic bronchitis is unfavourable but it is surprising what can be achieved by a short but vigorous course of pre-operative physiotherapy and dental toilet. Dyspnoea is no absolute contra-indication; it is a symptom of the disease and may improve after resection; nor are infection and pyrexia due to bronchial obstruction, for these can frequently be relieved by bronchoscopic clearance of the lumen before operation.

Paralysis of a vocal cord or the diaphragm does not always rule out resection, but obstruction of the superior vena cava almost certainly does. A lymphocytic effusion is no bar as it is due to secondary infection rather than pleural involvement by growth, but a bloodstained effusion usually rules out resection. The histology of the growth is ignored in assessing operability or prognosis, as some of the most satisfactory long term results have been achieved by resecting anaplastic oat-cell carcinomata.

### EFFECTS OF RESECTION

What of the functional results following resection? The disability is less than would be expected. The average patient retains about 75 per cent. of his exercise tolerance, can return to his previous employment and hobbies, and lead a useful and happy life. A few cases may be crippled by pulmonary insufficiency. Unfortunately, pulmonary function tests are not of great clinical value, and the ward sister is often the best judge of a patient's cardio-pulmonary reserve.

A third of the patients who die within five years of resection succumb to non-malignant conditions, such as right heart failure, contra-lateral pneumonia, or unrelated disease with no evidence of any recurrence of the growth at autopsy. Some of these patients might be salvaged with more adequate physiotherapy. In this series of 432 resections there has been no instance of a second primary growth developing in the remaining lung; possibly because the patients are advised to give up smoking! Multiple lung primaries are very rare and there have been only two definite cases of bilateral primaries amongst the 1,239 patients admitted for investigation. In the few reported cases both primaries have usually occurred in the same lung.

### EARLIER DIAGNOSIS

The main problem that worries the surgeon at present is how to achieve earlier diagnosis and earlier resection till such time as the Department of Preventive Medicine relieves him of the responsibility. The early symptoms are well recognised but their significance may be masked by their mildness. Radiological diagnosis is full of pitfalls. Any shadow can be caused by a growth and should be suspect till exonerated by complete investigation. The X-ray may be normal in the presence of advanced inoperable disease, and radiological improvement does not rule out cancer. There is no place for the prolonged observation of fascinating X-ray shadows, nor for the "come back in three months, for another X-ray" approach. Nor is there any place for the speculative course of antibiotics to see if the lesion will clear.

There are only two ways in which the diagnosis can be made with certainty: by bronchoscopy and by exploratory thoracotomy. A bronchoscopic biopsy is available in less than half the cases examined but the presence of a growth can be inferred with accuracy on collateral evidence in the majority, but it takes about 10 years of practice in correlating endoscopic and operative findings before a reliable opinion can be expressed. One encouraging feature of the present situation is the increasing number of negative bronchoscopies that are performed. A negative bronchoscopy however, does not rule out a growth, and in consequence these cases are followed for at least twelve months. Recently, three early and possibly curable growths have come to light during such a period of observation. It is unlikely that a nation of hypochondriacs will be created by adequate early investigation, because the public have now been conditioned to expect it. Patients are far more surprised when told they have not got cancer of the lung than when their suspicions are confirmed.

Exploratory thoracotomy is the final court of appeal in many cases, and there should be no hesitation in resorting to this method of diagnosis. Examination of a frozen section or a quick smear examination may assist the interpretation of operative findings. Nobody would wish to subject a patient to an unnecessary operation but it is surprising how seldom exploration fails to reveal a growth. The "negative thoracotomy rate" is shown in Table IV.

TABLE IV

*"Negative thoracotomy rate"*

On 614 explorations, cancer was proven and confirmed by histology. There were 16 resections for chronic pneumonitis and 6 negative explorations, no serious disease being found.

Most of the sixteen resections for "chronic pneumonitis" were lobectomies for irreparably damaged lobes, but a few patients have lost a lung when a lobectomy would have sufficed had the inflammatory nature of the lesion been recognised at operation. The incidence of wrong diagnosis at thoracotomy is now very low indeed.

Bronchial carcinoma may remain silent till it is incurable but in the majority of cases diagnosis and treatment are possible within 3 months of the onset of symptoms, with the facilities available at the present time.

Unfortunately there is still no real sense of urgency in the profession's approach to this problem. The figures in Table V (Thompson 1957) for a Metropolitan region speak

TABLE V

*Average delay*

(A Metropolitan Region)

| | | |
|---|---|---|
| Onset of symptoms to operation | = | 7·4 months. |
| Patient's delay | = | 1·5 months. |
| Professional delay | = | 5·9 months. |
| (G.P., Radiologist, Chest Physician, Chest Surgeon). | | |

TABLE VI

*Duration of symptoms*

*S.W. Region*

Symptoms present 3 months or longer

1950 : 69 per cent. of all cases

1955 : 70 per cent. of all cases.

for themselves, Mason has reported similar figures from the Newcastle region (Mason 1958). There is evidence that the position is more satisfactory in the Bristol area, but the figures in Table VI from the South-West Region afford no cause for complacency, and must be profoundly disappointing to all concerned in the battle against this disease. The present diagnostic procedure is cumbersome and inefficient. More concerted action is needed and there must be synchronous training of all available diagnostic and therapeutic missiles on the target. One of the more promising and fruitful of recent developments is the combined out-patient and bronchoscopy clinic with both chest physician and surgeon in attendance. Time is saved and the patient spared a second bronchoscopy.

Not until this disease is treated by all concerned as an acute surgical emergency will the results of treatment improve.

### REFERENCES

Thompson, V.C., N.A.P.T. Bulletin, 1957 **20** 2.
Mason, G. 1958 Personal Communication.

## Figures and Tables

**Figure f1:**
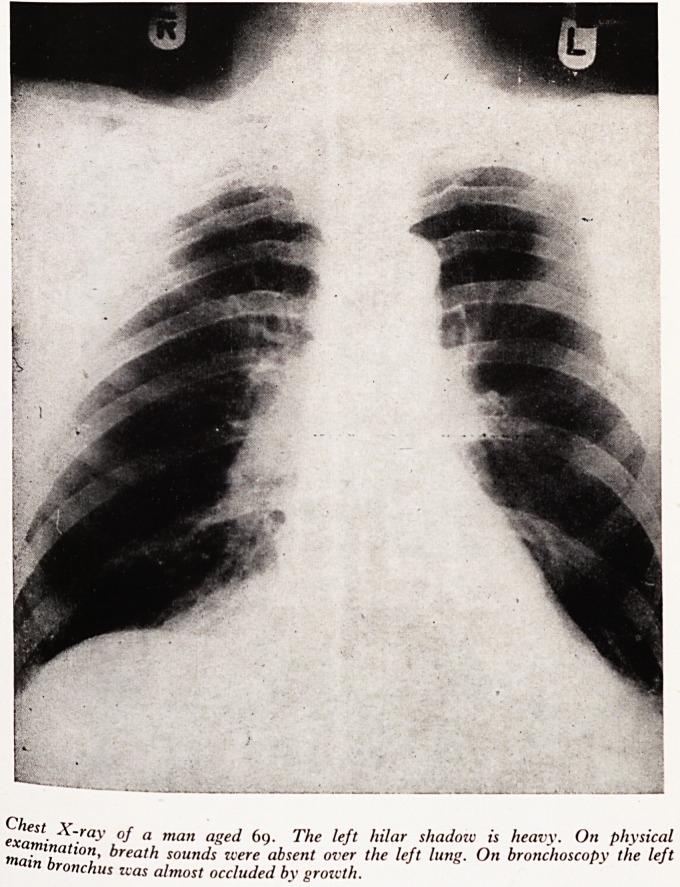


**(a) f2:**
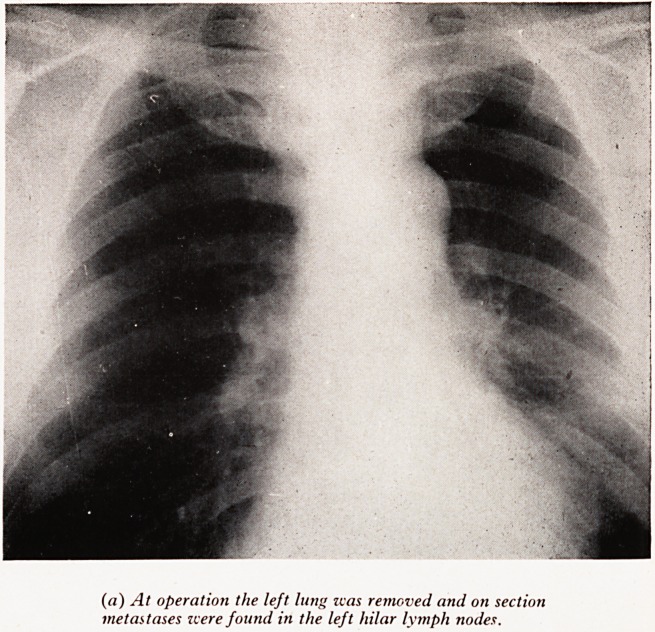


**(b) f3:**
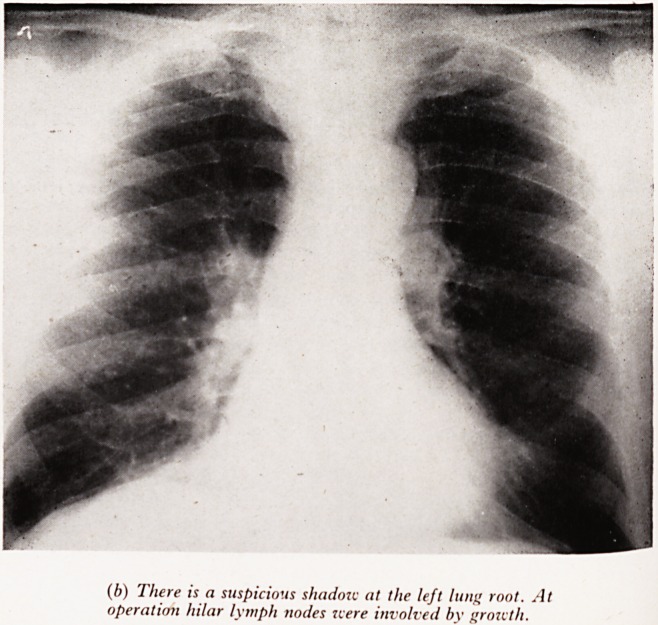


**(a) f4:**
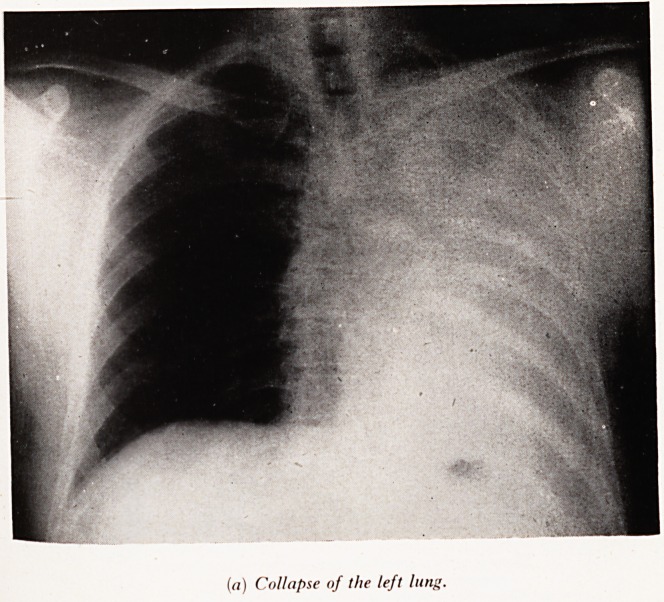


**(b) f5:**
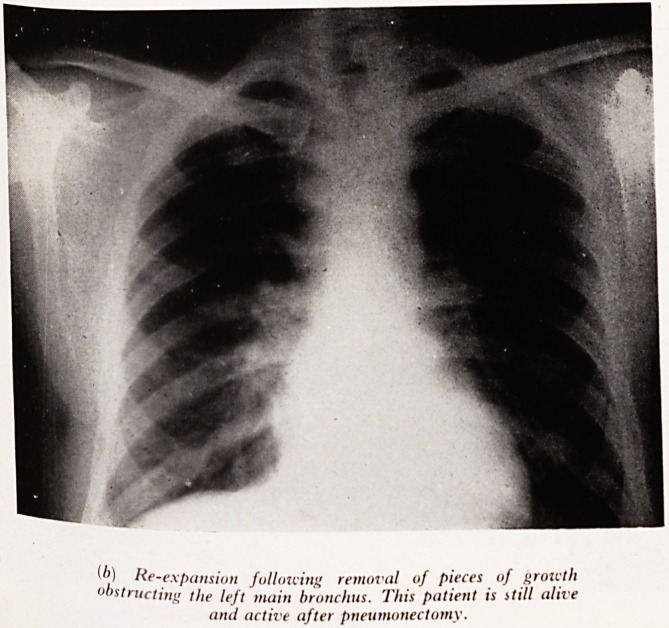


**(a) f6:**
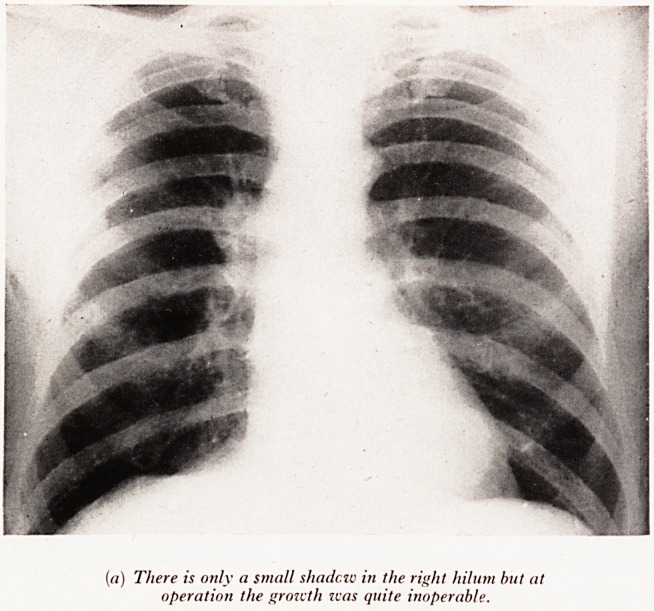


**(b) f7:**
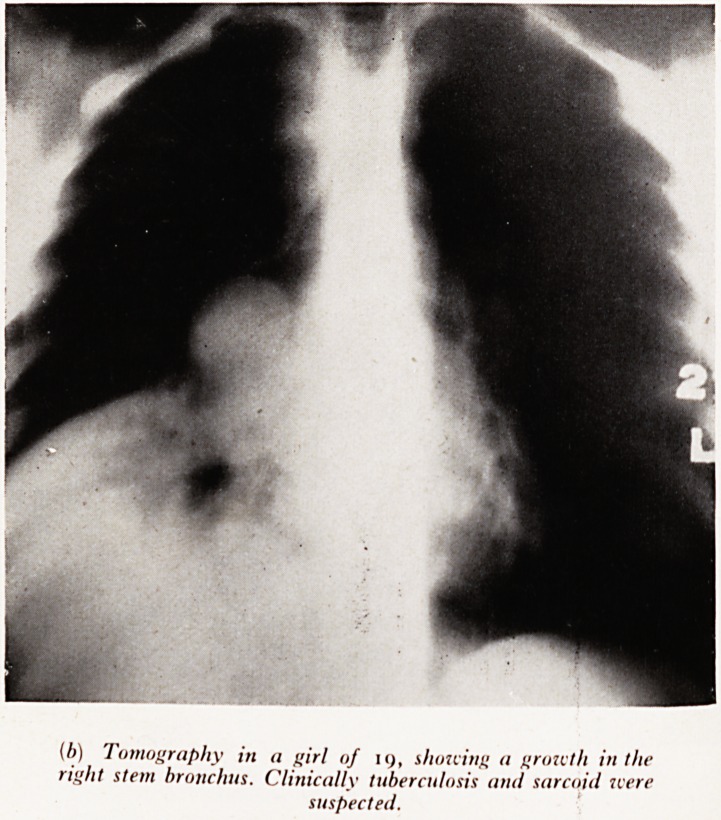


**(a) f8:**
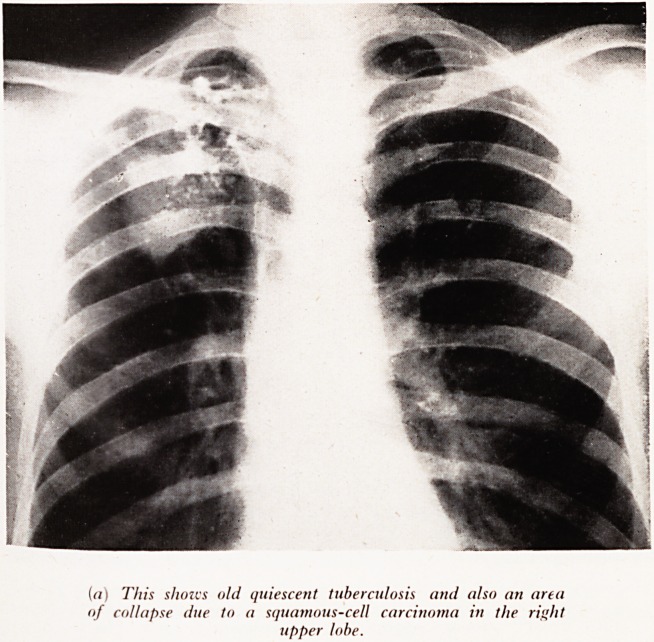


**(b) f9:**
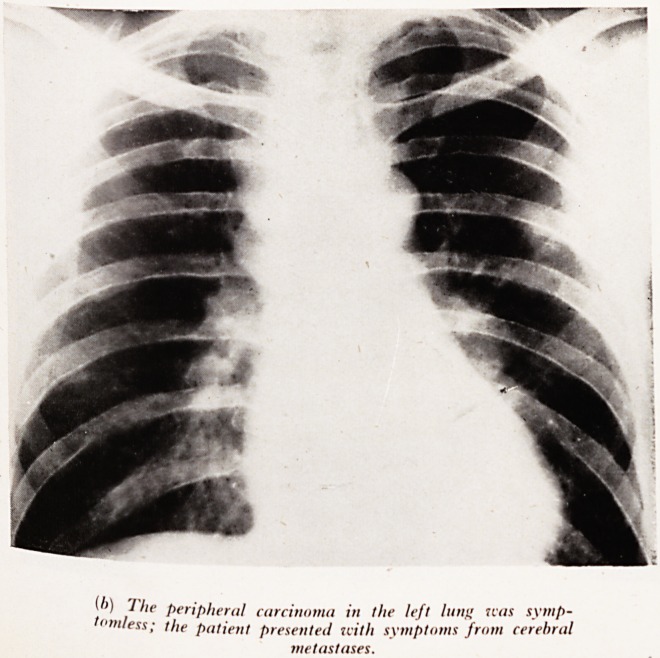


**(a), (b). f10:**